# ONT read assembly of the black rhino genome

**DOI:** 10.1186/s12863-024-01214-0

**Published:** 2024-03-05

**Authors:** Ken Kraaijeveld, Koen Bossers, Nikola Petrusevski, Stef Pieterman, Linda G.R. Bruins-van Sonsbeek, Floyd Wittink

**Affiliations:** 1 Leiden Centre for Applied Bioscience, University of Applied Sciences Leiden, Darwinweg 24, 2333CR Leiden, The Netherlands; 2 Rotterdam Zoo, 3000AM Rotterdam, PO Box 352, The Netherlands

## Abstract

**Objectives:**

The black rhinoceros (*Diceros bicornis*) is an endangered mammal for which a captive breeding program is part of the conservation effort. Black rhinos in zoo’s often suffer from chronic infections and heamochromatosis. Furthermore, breeding is hampered by low male fertility. To aid a research project studying these topics, we sequenced and assembled the genome of a captive male black rhino using ONT sequencing data only.

**Data description:**

This work produced over 100 Gb whole genome sequencing reads from whole blood. These were assembled into a 2.47 Gb draft genome consisting of 834 contigs with an N50 of 29.53 Mb. The genome annotation was lifted over from an available genome annotation for black rhino, which resulted in the retrieval of over 99% of gene features. This new genome assembly will be a valuable resource in for conservation genetic research in this species.

**Keywords:**

Black rhinoceros, *Diceros bicornis*, Genome assembly, Long reads, Whole genome sequencing.

## Objective

Ongoing habitat loss, overexploitation, climate change and other factors have severely reduced population size of many large mammals. In addition to preventing and reversing these causal factors of biodiversity loss, ex-situ captive breeding programs can be an effective method for boosting small populations. Captive breeding programs are being attempted to augment the wild population of several species and prevent their extinction [1, 2].

Genomic resources play an increasingly important role in conservation-related captive breeding projects [2]. For example, measurement of genetic diversity and relatedness between individuals may inform choice of breeding individuals [1]. Genomic resources may also aid in managing health and fertility of captive animals [2].

The Eastern black rhinoceros *Diceros bicornis* is critically endangered in the wild. The total number of individuals is estimated at 3,142, many of which live in captivity [3]. A captive breeding program aimed at boosting population size is currently ongoing [4]. Several aspects of this effort benefit from genomic resources, including genetics-informed choice of breeding animals, microbiome research in relation to health issues and the genetics of male fertility. Several genome assemblies are available for black rhino, but these are either from a female or produced using short reads, preventing the study of Y-chromosomal regions that may affect male fertility. We sequenced the genome of a male black rhino using ONT long reads with the aim of generating long contigs that can be assigned to the sex chromosomes.

We present here the results of the sequencing, assembly and annotation. A preliminary identification of sex-chromosomal regions in this genome assembly is outlined in [5].

## Data description

A blood sample was collected from the Eastern black rhino ‘Vungu’ held at Blijdorp Zoo (Rotterdam, The Netherlands) during routine medical examination. DNA was extracted from whole blood using the Nanobind CBB kit (Circulomics) following the manufacturer’s instructions. ONT sequencing libraries were prepared using ligation sequencing kit v9 (SQK-LSK109) and v10 (SQK-LSK110) and sequenced on a Minion device with both R9 and R10 flowcells. FAST5 data was basecalled using the Guppy basecaller version 3.3.3. This resulted in 5.7 million reads with an average read length of 18 kb (Table 1, Data set 1, [6]). The sequence reads were assembled *de novo* using Flye with the parameters --nano-raw = ONT regular reads, pre-Guppy5, −i 2 = number of polishing iterations [7]. This resulted in a draft genome assembly of 2.47 Gb, consisting of 834 contigs with a contig N50 of 29.5 Mb. The draft genome assembly was annotated by lifting over the annotation of accession GCA_013634535.1 [8], which is the only Black rhino genome assembly for which an annotation is currently available. Over 99.9% of gene features were retrieved in the new genome assembly.

**Table 1:** Overview of data files/data sets

Label	Name of data file/data set	File types (file extension)	Data repository and identifier (DOI or accession number)
Data file 1	Genome assembly of *D. bicornis*	fasta file (.fna)	https://www.ncbi.nlm.nih.gov/nuccore/JANTPW010000000 [9]
Data file 2	Gene annotation	gff file (.gff)	Figshare, 10.6084/m9.figshare.22699801 [10]
Data set 1	ONT sequence reads of *D. bicornis* genomic DNA	fastq files (.fastq)	NCBI Sequence Read Archive (https://www.ncbi.nlm.nih.gov/bioproject/PRJNA777872) [6]

## Limitations

The genome assembly is still fragmented and can be further improved using Hi-C, bionano or other techniques.

## Data Availability

The data described in this Data note can be freely and openly accessed on NCBI SRA under Bioproject ID PRJNA777872 [6], NCBI GenBank under accession number JANTPW000000000 [9] and figshare (10.6084/m9.figshare.22699801) [10]. Please see Table 1 for details and links to the data.

## Abbreviations

NCBI: National Center for Biotechnology Information
ONT: Oxford Nanopore Technologies
